# Antimicrobial resistance databases: opportunities and challenges for public health

**DOI:** 10.1038/s44259-025-00169-1

**Published:** 2026-01-08

**Authors:** Chad M. Centner, Sabrina Di Gregorio, Silvia Argimón, Alice Brankin, Anna Dean, Daniel Marcano Zamora, Silvia Bertagnolio

**Affiliations:** 1 Surveillance and Laboratory Strengthening, Department of Antimicrobial Resistance, World Health Organization (WHO), Geneva, Switzerland; 2 Public Health Laboratory Innovation Platforms, WHO Hub for Pandemic and Epidemic Intelligence, WHO Health Emergency Preparedness and Response Programme, Berlin, Germany

**Keywords:** Computational biology and bioinformatics, Health care, Microbiology

## Abstract

Antimicrobial resistance (AMR) databases enable the identification of AMR determinants from pathogen sequence data and the prediction of resistance profiles, enhancing AMR surveillance and informing a range of public health interventions. This review compares freely available and regularly updated AMR databases, explores their public health value and highlights key challenges to and opportunities for fully harnessing their potential.

## Introduction

Bacterial antimicrobial resistance (AMR) is one of the most significant challenges facing human health and requires urgent action^1^ Next generation sequencing technologies, including whole-genome sequencing (WGS), are increasingly used to monitor AMR and inform public health interventions, such as surveillance^2,3^, outbreak management^4^, infection prevention and control^5^, programmatic care^6^, and diagnostics development^7^. Genomic surveillance of AMR and knowledge of AMR determinants were identified as global strategic and research priorities^8–10^,including and rapid accurate methods to detect them^11^.

Resistance may be intrinsic (i.e. naturally found in a species) or acquired (e.g., though mutation or horizontal gene transfer). AMR determinants are genes and genetic modifications that confer resistance to one or more antibiotics and antibiotic classes. AMR genes encode proteins that modify the antibiotic (e.g., β-lactamases), modify and protect the antibiotic target site (e.g., ribosomal RNA methyltransferases), provide an additional target for which the antibiotic has reduced affinity (e.g., altered penicillin binding proteins), provide alternative metabolic pathways bypassing the antibiotic’s action (e.g., dihydrofolate reductases) or that extrude the antibiotic outside the cell (e.g., efflux pumps)^12,13^. Acquired AMR genes are often gained *en bloc* by horizontal gene transfer of mobile genetic elements that can move between DNA molecules, such as insertion sequences and transposons, or between bacterial cells, such as plasmids and integrative conjugative elements^14^.

Genetic modifications associated with resistance may change a single nucleotide (point mutations) or involve larger rearrangements (insertions or deletions) affecting either coding or regulatory regions. They may lead to alteration of the antibiotic target site (e.g., point mutations in *rpoB* leading to rifampicin resistance in *M. tuberculosis*), increased expression of an antibiotic target (e.g., point mutations in the promoter of *folA* encoding dihydrofolate reductase, the target of trimethoprim), or decreased antibiotic uptake (e.g., a small insertion in the *ompK36* porin gene, or an in-frame deletion in the *oprD* porin gene leading to decreased antibiotic permeability)^12,13^. Replacement of natural gene promoters with stronger promoters from insertion sequences via horizontal gene transfer also leads to resistance. For example, insertion of IS*Aba1* upstream of naturally-occuring *bla*_*ampC*_ in *Acinetobacter baumannii* leads to cephalosporinase overproduction and cephalosporin resistance^15^.

Databases of AMR determinants are curated repositories that store reference nucleotide and protein sequences of genes known to confer resistance, as well as wild-type genes or sequences that can confer resistance when modified. They also include associated information needed to identify these sequences in pathogen genomes, such as rules, computational models and classification hierarchies. As such, they need to capture the diversity of genes and genetic modifications associated with resistance. However, currently available databases vary widely in size, scope, structure, content, annotation, curation approach and update frequency^12,16–19^. Some are species-specific (Table 1), while others span multiple or all WHO bacterial priority pathogens^20^. Species-agnostic databases often operate under the assumption that determinants have the same effect across pathogens, which may not be the case. Databases also differ in the range of antibiotics and antibiotic classes they cover. They may comprise genes, mutations, or both, but their inclusion and exclusion criteria may also differ.

Bioinformatic tools compare pathogen sequences to those in AMR databases, detect and identify AMR determinants, and report them with additional information such as the associated antibiotic, the position of the determinant in the genome and sequence similarity (the percentage match between the pathogen and reference sequence). This information can then be used to predict phenotypic resistance. Bioinformatic tools employ a variety of computational approaches to detect AMR determinants in pathogen sequences, differing in reporting format and content^12,21–28^. Both database and associated tools may be accessed through command-line interfaces for integration into user bioinformatic workflows, or through web-based tools. Web-based interfaces are generally more user-friendly and accessible to those without prior bioinformatic expertise. They streamline analyses by automating workflows, use off-site computing resources, and produce reports with quick turnaround times. Additionally, several high-quality external bioinformatic tools are available that leverage one or multiple of these databases^12,17,18,24,25,29,30^.

## Methods

To identify AMR databases, we searched National Centre for Biotechnology Information (NCBI) PubMed using the term: (antimicrobial resistan* OR antibiotic resistan*) AND (mutation OR gene OR variant OR determinant) AND (catalogue OR database) for articles published between 1-January-2017 and 31-December-2023. We identified 1300 articles, of which 22 pertained directly to 12 AMR databases. Additional checks of reference lists and web searches pointed to an additional 12 databases. Of these 24 databases, 8 met these inclusion criteria: relevant for use with whole genome sequences (as opposed to metagenomes); free; regularly updated (defined as ≥1 update between 1-January-2024 and 1-January-2025); relevant to ≥1 WHO bacterial priority pathogen^20^; and spanning >1 AMR mechanism and/or >1 antibiotic and/or disinfection classes (Table 1).

**Table 1:** Regularly updated AMR databases

Database	Developer	Content type	Content class	Approximate number of genes and alleles	Curation strategy	Species-specific curation	Phenotype prediction	Latest Version	Approximate frequency of updates	URL	Associated bioinformatic tool	Reference
CARD	McMaster University	Proteins, genes and point mutations, ontologies for antibiotic resistance and detection models	Antibiotics, disinfecting agents and antiseptics	9600	Manual and computer-assisted (CARD-Shark) curation of literature, QC checks, community curation	Yes, for mutations in some species	No	Version 4.0.0 (18th December 2024)	1–3 months	https://card.mcmaster.ca/download	RGI (command-line and web-based)	^26^
AMRFinderPlus	National Center for Biotechnology Information	Proteins, genes and point mutations, reference gene hierarchy and reference hidden markov models	Antibiotics, stress (disinfecting agents and antiseptics, heat, metal), virulence	9900	Manual curation of literature searches, communication with experts, Lahey and Pasteur β-lactamase databases and CARD and ResFinder and PointFinder, automated QC checks	Yes, for mutations in some species	No	Version 4.0.1 (18th December 2024)	2-4 months	https://github.com/ncbi/amr/wiki/AMRFinderPlus-database	AMRFinderPlus (command-line based, Galaxy servers)	^23,34,35^
ResFinder	Technical University of Denmark	Genes, descriptive genotype to phenotype and species-specific bacterial resistance data	Antibiotics	3200	Manual curation of literature, CARD, ARDB^a^, BLDB and community curation.	Yes, for some species	Yes, for some species	Version 2.4.0 (13th December 2024)	1-5 months	https://bitbucket.org/genomicepidemiology/resfinder_db/src/master/	ResFinder (command-line and web-based)	^39^
PointFinder	Technical University of Denmark	Point mutations, descriptive genotype to phenotype and species-specific bacterial resistance data	Antibiotics	1300	Manual curation of literature, and community curation	Yes, for some species	Yes, for some species	Version 4.1.1 (8th August 2024)	3-5 months	https://bitbucket.org/genomicepidemiology/pointfinder_db/src/master/	ResFinder (command-line and web-based)	^40^
DisinFinder	Technical University of Denmark	Genes and descriptive data	Disinfecting agents and antiseptics	16	Manual curation of literature	No	No	Version 2.0.1 (31th May 2023)	5 months	https://bitbucket.org/genomicepidemiology/disinfinder_db/src/master/	ResFinder (command-line and web-based)	-
Kleborate	Monash University and The London School of Hygiene & Tropical Medicine	Genes, point mutations, gene truncations and intrinsic β-lactamases	Antibiotics	2700	Manual curation of CARD v3.2.9 and ARG-ANNOT^a^ databases	Yes	No	Version 3.1.2 (30th October 2024)	1–4 months	https://github.com/klebgenomics/Kleborate	Kleborate (command-line, Galaxy servers)	^27^
Pathogenwatch	University of Oxford	Genes and point mutations, interactions	Antibiotics	700	Manual curation of literature searches, personal communications, and CARD, ResFinder, AMRfinderplus and Kleborate databases	Yes	Yes	Version 23.1.0 (13th November 2024)	1–2 months	https://github.com/pathogenwatch-oss/amr-libraries https://pathogen.watch	Pathogenwatch (web-based)	^47,49,50^
WHO catalogue of mutations in MTBc and their association with drug resistance	WHO	Point mutations, associations with resistance	Antibiotics	48,152	Standardized bioinformatic analysis of genomes with matched AST results, followed by an association study and confidence grading method	Yes	Yes	2nd Edition (2023)	7–12 months	https://tbsequencing.who.int/mutations	None	^51,54^

Information correct as of 1st January 2025.

*ARDB* Antibiotic Resistance Genes Database, *ARG-ANNOT* Antibiotic Resistance Gene-ANNOTation, *AST* antimicrobial susceptibility testing, *BLDB* Beta-Lactamase Database, *CARD* Comprehensive Antibiotic Resistance Database, *QC* quality control, *MTB*c *Mycobacterium tuberculosis* complex, *RGI* resistance gene identifier, *WHO* World Health Organization.

^a^Database no longer actively maintained.

## Review of current databases

### Overview

Other reviews have previously documented AMR databases^12,17,18^, either in the broader context of genomic methods and resources to study and monitor AMR or focussing on their gene content. Here, we compare and contrast AMR databases (Table 1) identified through literature and web searches and meeting pre-specified criteria, while providing a structured critical analysis framed from public health and policy perspectives.

### Comprehensive antibiotic resistance database (CARD)

CARD includes a curated sequence database built on a structure known as the antibiotic resistance ontology (ARO) and a set of AMR detection models. The sequence database comprises AMR genes and mutations (not all clinically relevant), and biocide resistance genes.

The ARO refers to controlled vocabularies, categories and formal definitions indicating the relationships between database elements. Each AMR determinant sequence is classified by an ontological path, comprising categories by gene family, resistance mechanism, drug class and affected antibiotic^26,31^. In particular, the classification of β-lactam antibiotics also includes functional groups: monobactams, penicillins, cephalosporins (1^st^-5^th^ generations), carbapenems and revised β-lactamase inhibitor combinations. The ARO is a key advantage of CARD; it improves data management, standardization, interoperability and quality of data, and contributes to consistent interpretation and genotype-phenotype associations.

**Fig. 1:**
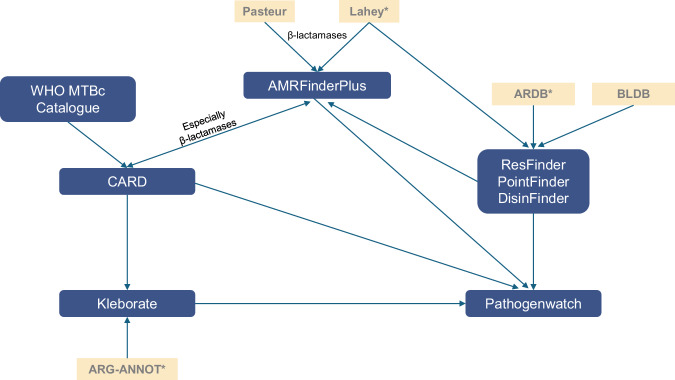
Data sharing between AMR databases. Blue blocks are databases reviewed in this article. Arrows indicate the general flow of information, including both historical data inputs and ongoing exchanges between databases. ARDB Antibiotic Resistance Genes Database; ARG-ANNOT Antibiotic Resistance Gene-ANNOTation; BLDB Beta-Lactamase Database; CARD Comprehensive Antimicrobial Resistance Database; MTBc *Mycobacterium tuberculosis* complex. *database no longer actively maintained. Up to date as of 1st January 2025. Lahey is one of the first beta-lactamase databases and has been used to inform many subsequent ones such as BLDB and ARG-ANNOT but has migrated to NCBI.

CARD uses a variety of detection models to identify AMR determinants from sequences, based on manually curated similarity cut-offs and information about molecular interactions^26,31^. For an AMR determinant to be included in CARD, it must be described in a peer-reviewed scientific publication and have its DNA sequence available in GenBank, along with clear experimental evidence of elevated MIC over controls (except β-lactamases)^26,31^. The database is manually curated and updated, aided by machine learning methods (CARD-Shark)^32^ and curation quality control algorithms. Curation is done through cross-database harmonization (Fig. 1), and the contribution of external researchers, software developers and others via the AMR curation public repository^33^, also done by AMRFinderPlus^34^.

The core CARD database is complemented by CARD-R, which stores AMR determinants not yet validated by clinical or experimental data –i.e. that were predicted in silico to confer resistance. CARD-R provides additional information on AMR determinants in public genomes, such as species and their location on chromosomes or plasmids^26^. CARD also features catalogues of AMR determinants specific to fungi (FungAMR) and *M. tuberculosis* (TB Mutations).

### AMRFinderPlus

The AMRFinderPlus database has three components: the reference gene catalogue, the reference hidden Markov models catalogue (hidden Markov models are a set of probabilistic models used to identify patterns within sequences and classify them into AMR protein families using manually curated and validated cut-offs^34^) and the reference gene hierarchy^23,34,35^. The reference gene catalogue is a curated database comprising AMR genes, point mutations and their associated proteins (core catalogue), along with additional stress- and virulence-related genes and proteins (plus catalogue). The reference gene hierarchy is an ontology structure that organizes and relates hidden Markov models to AMR determinants and classifies them by antimicrobial class (e.g., β-lactam), subclass (e.g., carbapenem), similarity and function.

Each protein and protein family included in the database is assigned a name and a gene symbol, based on the internal hierarchy, cut-offs and rules^34^. Each gene symbol can then be associated with related protein sequences (e.g., *bla*_*KPC*_), and each allele with a single amino acid sequence variant (e.g., *bla*_*KPC-2*_)^34^. This standardized approach is key to enhance identification and naming of detected determinants and assigning new alleles (done by NCBI for β-lactamases^36^, Qnr and MCR alleles).

AMRFinderPlus gathers determinants via literature surveys, data exchange with external curated sources and other mechanisms^34^. Inclusion of a determinant requires experimental verification or a highly similar hit to an experimentally verified protein, but supporting evidence in the literature is not required for allele assignment^34^. AMRFinderPlus established different curation approaches for genes, alleles, point mutations and hierarchy levels, with most being manual. It also applies automated quality-control checks to the sequences that are deposited in the database and for resolving nomenclature conflicts^34^.

AMRFinderPlus is used in NCBI’s Pathogen Detection pipeline^23,34,35^, providing results to the National Database of Resistance Organisms (NDARO) and its associated isolate browser, antimicrobial susceptibility testing (AST) browser, and databases of microbial genetic and genomic elements (MicroBIGG-E) and geographical distribution/coverage by species (MicroBIGG-E Map)^37^.

### ResFinder

ResFinder contains sequences of AMR genes^22,38,39^ and its associated database, PointFinder, contains information about chromosomal point mutations conferring resistance in selected bacterial species^22,38–40^. The primary focus has been to only include acquired AMR determinants. Additionally, DisinFinder –containing acquired disinfectant resistance genes– complements both AMR databases. ResFinder is supplemented by ResFinderFG which comprises AMR genes identified through functional cloning^41^. ResFinder initially drew on Antibiotic Resistance Genes Database (ARDB)^42^, and the Beta-Lactamase DataBase (BLDB)^43^ as data sources, but is currently manually curated by reviewing published literature^38,39^. Communication with researchers ensures that new AMR determinants meet requirements for inclusion^22^.

In addition to the focus on acquired resistance, another key difference with other databases is that the curation is focused on the in silico prediction of clinically relevant phenotypic antibiotic resistance profiles for selected bacterial species. For this purpose, ResFinder utilises genotype-to-phenotype tables for various antimicrobial classes and compounds, and species-specific panels for in silico antibiograms. The ResFinder software detects the presence of genetic determinants from all three databases and interprets specific combinations of multiple mutations or of genes and mutations that are needed to cause resistance in select species^22,39^.

### Kleborate

Kleborate is a tool for the analysis of *Klebsiella pneumoniae* species complex genomes that performs quality control (QC), taxonomic assignment, multi-locus sequence typing (MLST), K and O locus typing, and detection of AMR determinants, virulence and serotype. The Kleborate AMR database is a species complex-specific database comprising intrinsic β-lactamase genes, horizontally acquired genes, point mutations, and gene truncations, as well as their associated antibiotic classes^27,44^. It was initially developed from a subset of AMR determinants found in CARD^27^ which were manually curated via exclusions, gene name changes, sequence replacements and additional inclusions of genetic resistance signatures from the Antibiotic Resistance Gene (ARG)-ANNOTation database^45^. The β-lactamases were classified by their enzyme activity (e.g., extended-spectrum β-lactamase, carbapenemase) and whether it is abolished by β-lactamase inhibitors^43,45^. The *bla*_*SHV*_ alleles were further curated through the analysis of high-quality paired genotype and phenotype data and a literature systematic review of experimental evidence. This allowed the assignment of *bla*_SHV_ alleles to functional classes associated with intrinsic or acquired resistance phenotypes (i.e., wild type resistance to ampicillin, extended-spectrum β-lactam resistance, or inhibitor-resistant β-lactam resistance), and the reclassification of alleles present in the NCBI reference gene catalogue and BLDB^44^.

AMR determinants included in the database must have associated resistance phenotypes with confirmed clinical relevance in published data. Unlike in other AMR databases, by providing a comprehensive classification and curation of AMR determinants Kleborate allows more detailed predictions of resistance in the *K. pneumoniae* complex. Understanding the resistance genotype is important as it determines the most appropriate antibiotic combinations to treat multidrug-resistant *K. pneumoniae* infections^46^.

### Pathogenwatch

Pathogenwatch is a web platform for genomic surveillance. Through a web interface accessible to users with different bioinformatic expertise, Pathogenwatch provides QC, taxonomic assignment, MLST, serotype, AMR genes and mutations, AMR phenotype, plasmid replicon types, and trees showing genetic relatedness among genomes for select species.

Pathogenwatch AMR databases are curated for *Campylobacter* spp., *Klebsiella* spp., *Neisseria gonorrhoeae*, *Staphylococcus aureus*, *Salmonella* Typhi*, Streptococcus pneumoniae*, *Vibrio cholerae* and *Enterococcus faecium*^28^. Where available, Pathogenwatch uses existing, community-endorsed taxon-specific databases, such as for *Klebsiella* spp. (Kleborate)^27,47^ and *E. faecium*^48^. The remaining taxon-specific databases are developed and manually curated by compiling evidence from the literature, input from international consortia^49,50^, personal communications and other databases such CARD, ResFinder and AMRFinderPlus. To reduce false positive predictions, each gene or mutation is verified using available experimental resistance data and genome sequence data before incorporation into the final library. Like ResFinder, Pathogenwatch also predicts species-specific antibiotic susceptibility profiles, including resistance conferred by the presence of a combination of AMR determinants, plus inducible resistance, decreased susceptibility (intermediate resistance) and genetic elements that modify AMR determinants to restore susceptibility (e.g., deletions or disruptions by insertion elements). Like Kleborate, Pathogenwatch generates a QC report to ensure that AMR predictions are only considered for high-quality genomes.

## WHO catalogue of mutations in *Mycobacterium tuberculosis* complex (MTBc) and their association with drug resistance

The WHO MTBc catalogue was developed through a systematic analysis of over 38,000 MTBc isolates from 45 countries. The data comprised high-quality phenotypic AST results for 13 anti-tuberculosis drugs matched with high-quality whole-genome sequences provided by several consortia and open data calls^51,52^. “Solo” mutations, i.e. single mutations within a set of genes of interest that best explain the observed drug resistance phenotype, were identified. The genotype-phenotype association was statistically evaluated, which enabled the calculation of odds ratios, positive predictive values, and confidence intervals to determine the certainty of the association with resistance. This enabled classification of mutations into five categories: associated with resistance, associated with resistance interim, uncertain significance, not associated with resistance, not associated with resistance interim. Interim categories reflect some uncertainty and they can be revised as new evidence becomes available.

The catalogue is released as a spreadsheet and variant call files (containing mutations, their genomic coordinates and associated antibiotics)^53^, with accompanying publications^51,52,54^. Independently developed bioinformatic tools, such as the resistance gene identifier (RGI)^26^, Mykrobe^55^ and TB Profiler^56^ use the catalogue to predict resistance from sequence data. The second edition of the catalogue increased the number of isolates and country representation, resulting in the addition of mutations associated with resistance to newly endorsed or repurposed drugs (e.g., bedaquiline, delamanid and linezolid)^54^.

## Value and opportunities for public health

AMR databases can support public health activities directed to reducing the burden of antibiotic-resistant infections^2,12,16^ (Box 1). They allow detection of all known AMR determinants from sequence data, enabling more specific surveillance than phenotype alone^2,16^. AMR databases can capture and catalogue rare or emerging resistance variants—such as polymerase chain reaction (PCR)-positive *bla*_*KPC*_ alleles with novel mutations that cause unusual or unexpected phenotypic resistance^16,57^—offering valuable insights into resistance mechanisms when conventional phenotypic and molecular tests are unavailable, unreliable or insensitive^16,57^. They can also facilitate the study of resistance determinant evolution and their geographical spread. Genomic surveillance in the Philippines found a nationally circulating clone carrying *bla*_*NDM-1*_ and *bla*_*NDM-7*_ on distinct plasmids, showing local and regional spread. Additionally, a hospital outbreak driven by a plasmid (IncFII) carrying the *bla*_*NDM-1*_ was spread across multiple genetic lineages in a neonatal intensive care unit^3^. AMR databases enable surveillance of the resistome through more recent approaches like untargeted or targeted metagenomic sequencing to understand how AMR determinants spread and evolve across clinical and environmental sources (e.g., wastewater surveillance)^58,59^. They have also supported shotgun proteomics for detecting AMR gene products^60^.

AMR databases play an important role in guiding the development of interventions, such as new diagnostic tests. These may include rapid tests such as PCR and loop-mediated isothermal amplification (LAMP) assays of particular utility in settings where conventional phenotypic tests or advanced testing such as panel-based PCR and genomics are unsuitable or still unattainable^7,61–63^. Notably, the WHO MTBc catalogue underpinned new WHO recommendations on the use of targeted next-generation sequencing (tNGS) tests for the diagnosis of drug-resistant tuberculosis as an alternative to conventional phenotypic methods^64^. By systematically documenting resistance-associated mutations, the catalogue harmonized the detection and interpretation of resistance across various drugs^51,52,54^ contributing to tNGS adoption, for example in Namibia^6^.

AMR genes can disseminate independently of host species through horizontal gene transfer and these events may remain undetected where surveillance relies on conventional speciation and phenotypic susceptibility testing alone^16,65^. Genomic surveillance revealed that a hospital outbreak was driven by a plasmid (IncFII) carrying *bla*_*NDM-1*_ spread across multiple genetic *K. pneumoniae* lineages in a neonatal intensive care unit^3^. AMR databases can facilitate prospective detection and timely response to outbreaks through identification of epidemiologically relevant determinants. Infection prevention and control measures can then be directed towards the specific resistance mechanism. Genomic surveillance of *Pseudomonas aeruginosa* ST621 revealed a decades-long hospital outbreak that persisted through sink-drain reservoirs and evolved multidrug resistance, but targeted infection-control interventions informed by these genomic findings ultimately suppressed transmission and ended the outbreak^4^. Databases of biocide resistance determinants (Table 1) can further support infection prevention and control measures^5,23,26^.

An understanding of specific AMR determinants in clinical specimens can guide patient care by informing treatment guidelines specific to the local context and epidemiology. For example, knowledge of certain *rpoB* mutations can guide the clinical management of rifampin-resistant TB by identifying rifabutin susceptibility and prompting testing for other second-line drugs^54^. Similarly, the treatment of carbapenemase-producing gram-negative organisms depends on the underlying carbapenemase, as certain β-lactam antibiotics have activity against specific carbapenemases^46^.

Box 1 Opportunities and challenges for AMR databases

**Table:** 

OPPORTUNITIES	**Description**
Enhanced surveillance	Enables detection of all known AMR determinants across sequenced isolates, offering greater specificity than phenotype alone for local-to-global surveillance.
Proactive outbreak detection and control	Facilitates early identification of epidemiologically-relevant resistance determinants, supporting timely, mechanism-specific responses.
Tailored infection prevention strategies	Supports the identification of biocide and resistance genes that may influence disinfection efficacy and IPC measures.
Accelerated diagnostic innovation	Guides the development of molecular assays (e.g., PCR, LAMP) and next-generation sequencing diagnostics, especially valuable where WGS is not feasible.
Evidence-based treatment	Enables resistance mechanism-informed clinical decisions, such as Ambler class-based therapy for carbapenemase-producing organisms.
Predictive modeling for resistance	Lays the foundation for machine learning and AI-driven prediction models, with potential to estimate resistance phenotypes and confidence levels.
Support for global initiatives	Provides a backbone for initiatives like WHO’s GLASS to harmonize genomic AMR surveillance, catalyzing norm-setting and global data sharing.
CHALLENGES	**Description**
Incomplete coverage of AMR diversity	Databases reflect known determinants, often derived from high-income settings, limiting representativeness and missing region-specific variants.
Accuracy of genotype-phenotype links	Misleading associations can arise from the inclusion of intrinsic, non-contributory AMR determinants or not considering complex genetic mechanisms.
Curation burden and inconsistencies	Manual expert curation is critical but slow and inconsistently applied across databases, risking outdated or unevenly validated content.
Fragmentation and duplication	Multiple overlapping databases vary in scope, structure, and update cycles, impeding integration and consistent interpretation.
Lack of standardization	Disparate naming conventions, gene-allele distinctions, and ontology structures hinder cross-database harmonization and data sharing.
Update and version control	Lagging updates and use of legacy versions in pipelines can lead to misinterpretation and reduced reliability of results.
Usability barriers	Many tools require bioinformatics expertise; while web interfaces improve access, they often lack flexibility and scalability for larger datasets.

*GLASS* Global Antimicrobial Resistance Surveillance System, *IPC* infection prevention and control, *LAMP* loop-mediated isothermal amplification, *PCR* polymerase chain reaction, *WGS* whole-genome sequencing, *WHO* World Health Organization.

## Challenges

Pathogen genomics is transforming public health efforts to combat AMR. Decreasing costs have enabled wider adoption, and ongoing advances in sequencing technologies and analytical tools are poised to further enhance its impact. However, realizing the full potential in the areas described above requires addressing several challenges, including those limiting the accurate prediction of resistance phenotypes from genomic data. AMR databases are a central component of these workflows and here we focus on the challenges related to their completeness, accuracy, standardization, timeliness and accessibility.

### Completeness

**Fig. 2:**
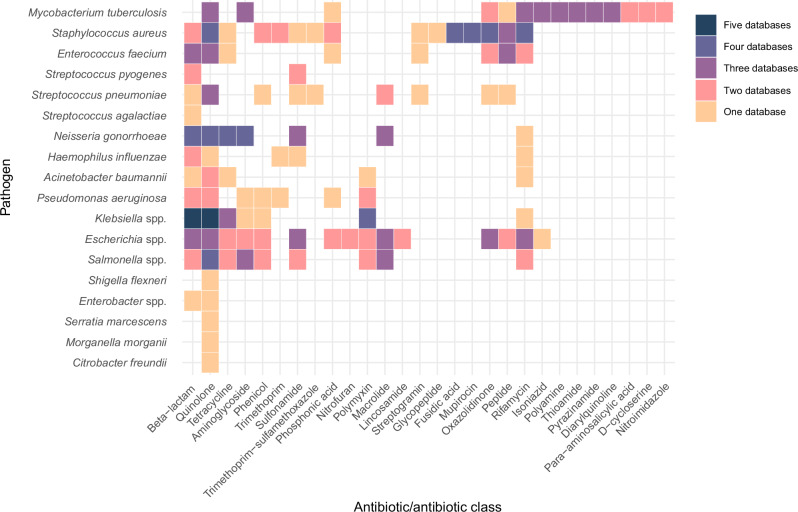
Heatmap illustrating the differential coverage of antimicrobial resistance (AMR) databases for point mutations associated with resistance across bacterial pathogens and antibiotics/antibiotic classes. Rows represent pathogens within the WHO bacterial pathogen priority list, while columns represent antibiotic/antibiotic classes. Colour shading indicate the total number of AMR databases that provide information on point mutations for each pathogen-antibitioc/antibiotic class combination (yellow = 1, pink = 2, violet = 3, blue = 4, dark blue = 5 databases [AMRFinderPlus, Kleborate, Pathogenwatch, PointFinder, Comprehensive Antimicrobial Resistance Database (CARD)]). Up to date as of 1st January 2025.

Completeness refers to the extent to which all required AMR determinants and associated information are present, but this depends on the specific purpose or purposes the database serves –for example, clinical resistance prediction versus high-resolution molecular epidemiology. Despite collaboration and data sharing (Fig. 1), AMR databases differ in size, age, scope, structure and purpose (Table 1). Consequently, different databases contain overlapping but different sets of AMR determinants for different pathogens and antibiotics or antibiotic classes^35^ (see Fig. 2 for a comparison of mutation coverage). For example, CARD collects AMR determinants regardless of their clinical relevance, while ResFinder focuses primarily on clinically-relevant ones. The WHO MTBc catalogue, Kleborate and Pathogenwatch are curated to predict species-specific AMR, while PointFinder, CARD and AMRFinderPlus do this for point mutations, but not necessarily for all antibiotics. This variation in AMR databases and tools not only reflects the complexity of bacterial AMR but also demonstrates fragmentation between databases and the duplication of efforts. Understanding the suitability and limitations of different AMR databases for pathogen and antibiotic combinations is essential for using and interpreting their outputs for different purposes.

Completeness is challenged by our insufficient understanding of the molecular mechanisms of resistance –AMR databases can only catalogue known determinants. AMR databases to date have been mostly developed using data from high-income settings, limiting global representativeness^66^. Local and regional variation in AMR determinants associated with specific resistance phenotypes, coupled with a lack of contextual information (e.g., geographical origin, treatment outcomes), can hinder the identification of uncharacterized genes, alleles, or mutations and affect downstream interpretation. For example, in a study from Valencia (Spain)^67^, and during the development of the second version of the WHO MTBc catalogue^54^, the analysis of *M. tuberculosis* genomes not included in the first version revealed relevant mutations that were previously unidentified. This highlights the value of large collections of well-characterized and genetically diverse high-quality genomes from diverse sources and regions (with matched AST phenotypes) to improve the completeness of AMR databases.

### Accuracy

Accuracy refers to the correct association between AMR determinants and the inferred resistance phenotype. Databases providing detailed ontological and hierarchical relationships between AMR determinants, antibiotics and phenotypes, such as CARD and AMRFinderPlus, allow a structured way to analyse and interpret data^23,26^. However, it is broadly assumed that the presence of a resistance gene or mutation directly impacts the resistance phenotype to a particular drug or drug class, because of the strong genotype-phenotype concordance found for some species and antimicrobial classes^48,49,51,68,69^. AMR databases often include determinants whose presence alone does not necessarily confer clinically relevant resistance, such as some species-specific, intrinsic AMR genes (Table 1)^27,51^. For example, chromosomal *bla*_*OXA-51-like*_ genes are ubiquitous in *A. baumannii* and encode weak carbapenemases which do not alter treatment options^70^. Differences between databases in cataloguing and reporting this additional information (clinical relevance, intrinsic resistance and species background) may affect interpretation and lead to inaccurate resistance phenotype predictions.

Moreover, databases do not completely cover the biological complexity of AMR in different species. The phenotypic expression of resistance can be affected by mobile genetic elements, mechanisms of gene regulation, changes in promoters/silencers, mutations in other intergenic regions, de novo ribosomal RNA mutations, increases in gene copy number and the additive effect of different AMR mechanisms and their interactions^13^. For example, the performance of CARD predictions for aminoglycoside and fluoroquinolone compounds in *E. coli* improved when compared to ResFinder, after exclusion of predictions of resistance based on determinants linked to efflux pump genes, whose expression is greatly influenced by transcription regulation^71^. So the mere presence of an AMR determinant does not guarantee expression sufficient to confer resistance detectable by standard phenotypic methods. Furthermore, differences in interpretive thresholds, such as epidemiological cut-offs versus clinical breakpoints (e.g., CLSI or EUCAST) affect the perceived concordance between genotypic and phenotypic resistance^72,73^.

Expert curation and the creation of expert rules^72,73^ are needed to improve the accuracy of genotype-phenotype associations in AMR databases, as demonstrated for *Klebsiella* spp.^27^, *M. tuberculosis*^51^, *E. faecium*^48^, *Pseudomonas aeruginosa*^74^ and *Salmonella* Typhi^75^. However, species-specific curation is time-intensive and particularly challenging for rarer and fastidious organisms where genotypic and phenotypic data are limited, such as *Haemophilus influenzae*^76^. In addition, identifying “solo” determinants, the basis of the genotype-phenotype associations in the MTBc catalogue, is considerably more challenging in organisms where resistance genes are frequently acquired *en bloc* through horizontal gene transfer.

### Standardization

The absence of global unified standards for naming and identifying resistance genes and alleles hinders comparison and poses a significant challenge to local-to-global genomic surveillance of AMR^77,78^. Even with consistent content, databases may differ in gene and allele naming –either when different names are assigned to the same gene or protein, or when one database classifies a sequence as a gene and another one as an allele^35^. Older databases may not use the most up-to-date methods for curation and annotation.

Efforts to standardise nomenclature^36,44^, data structures, ontologies and hierarchies^23,26^ and harmonize gene name conventions between databases^78,79^ are important steps towards developing unified standards for more effective comparisons and data exchange. CARD and AMRFinderPlus participate in public discussion of nomenclature issues at the arpcard/amr_curation GitHub site^80^. Equally important are initiatives to standardize genomics workflows^77^, such as the ISO-certified AbritAMR^30^, and harmonization of AMR reports in a single consistent format^81^.

### Timeliness

Regular database updates and revisions are crucial for timely inclusion of newly discovered AMR determinants and improved annotations, such as the reclassification of *bla*_*SHV*_ alleles by Kleborate^44^. New versions are often released when new evidence requires substantial additions or revisions (Table 1). Manual curation of peer-reviewed experimental evidence ensures rigorous evaluation, but is labour-intensive, time-consuming and potentially subjective. As the number of publications grows^16^, manual curation and regular updates become increasingly time- and resource-consuming. Some database developers have addressed these challenges through machine learning methods to optimize literature review^32^ and the use of quality control algorithms^34^. Standardized data-driven approaches to curation would allow frequent analyses of large datasets; however, curation would still rely on human expertise^82,83^.

When databases are updated, the many bioinformatic pipelines implementing local copies also need updates. Despite database versioning –i.e. the release of discrete snapshots of a database over time with tracked changes^51,54,84^, outdated legacy databases and tools are still available online and their use may lead to inaccurate results.

### Accessibility and usage

Choosing the most appropriate AMR database (Table 1), prediction tool, and parameters requires understanding of the available options and bioinformatics expertise, which may vary across different settings. Additionally, parameters used can significantly affect whether genes are matched from query sequences to databases, and ideal parameters for gene detection may change based on bacterial species and subtype^85^. To address this, AMR databases and their associated bioinformatic tools are available through a variety of web-based interfaces, which makes them well-suited for rapid response in resource-limited settings. However, they often provide less capacity to process large datasets and less customization compared to command-line tools.

## Conclusion and future perspectives

This review highlighted the variability in AMR databases as well as their growing significance for public health. They play important roles in genomic surveillance, outbreak management, and infection prevention and control. To realize the full potential of AMR databases to enable these interventions, collaborative efforts should focus on improving data quality, standardization, global representativeness, and accessibility while avoiding duplication and fragmentation. The WHO MTBc catalogue is a powerful example of the impact of a community-driven, comprehensive, standardized catalogue, and similar initiatives are needed for all priority pathogens^9,10^.

WHO currently hosts the Global Antimicrobial Resistance Surveillance System (GLASS), which in the coming years will integrate genomic data for enhanced local-to global surveillance and situational awareness. GLASS will need centralized resources for AMR genomic data, including tools, guidance and a catalogue of AMR determinants. As a centralized, comprehensive resource, the catalogue may initially focus on selected priority pathogens and clinically significant determinants to support global surveillance. Compiled through collaboration and leveraging the many existing initiatives outlined in this review, the catalogue may evolve to include data from diverse settings, ensuring global representation. International collaboration and data sharing, according to data sharing principles^86,87^, will be vital for curating and harmonizing AMR databases with increased completeness, coverage and representation.

Future iterations may incorporate statistical models with certainty estimates to support clinical care. The integration of genomic and phenotypic data will be essential for developing models to predict phenotypic resistance from genome sequences^44,72,82,83,88,89^. Machine learning methods and artificial intelligence are poised to improve phenotype prediction by identifying low-certainty calls^26,37,44,51,52^, novel AMR mechanisms, and interactions between mechanisms^90^. Large, high-quality datasets spanning different sources and regions, and encompassing a wide range of pathogen lineages, will be needed to inform robust models^91,92^. Implementation of data standards and quality control will ensure validity of the catalogue^51,54,93–98^.

Centralized resources accessible to users with varied expertise will ensure that public-health benefits are equitably distributed, including the identification of geographically relevant targets for drug, diagnostics and vaccine development. To accelerate progress, we call on governments, public health agencies, researchers and funders to commit sustained support for building and maintaining standardized AMR genomic resources, a global priority^8–10^. This ambitious vision promises to significantly advance our understanding and management of AMR on a global scale.

## Data Availability

The datasets and code used to construct Table 1 and Fig. 2 can be accessed here: https://worldhealthorg-my.sharepoint.com/:f:/g/personal/centnerc_who_int/IgC8e2i67D5yQrzOXxD2ChKPARK2uRA8K8CqXHIxd26fdSI?e=DHahWP. They were generated using datasets obtained from CARD [https://card.mcmaster.ca/download]; AMRFinderplus [https://github.com/ncbi/amr/wiki/AMRFinderPlus-database]; ResFinder [https://bitbucket.org/genomicepidemiology/resfinder_db/src/master/]; PointFinder [https://bitbucket.org/genomicepidemiology/pointfinder_db/src/master/]; Kleborate [https://github.com/klebgenomics/Kleborate], Pathogenwatch [https://github.com/pathogenwatch-oss/amr-libraries]; and the WHO MTBc catalogue [https://github.com/GTB-tbsequencing/mutation-catalogue-2023/tree/main/Final%20Result%20Files] repositories.
